# Prevalence of Verotoxin-Producing *Escherichia coli* (VTEC) in a survey of dairy cattle in Najaf, Iraq

**Published:** 2010-09

**Authors:** A Al-Charrakh, A Al-Muhana

**Affiliations:** 1 College of Medicine, Babylon University, Department of Microbiology, College of Medicine, Babylon University; 2 College of medicine, Kufa University, Microbiology

**Keywords:** Verotoxin-producing *E. coli*, prevalence, O157:H7 serotype, dairy cattle

## Abstract

**Background and Objectives:**

Dairy cattle have been implicated as principal reservoir of Verotoxin-Producing *Escherichia coli* (VTEC), with undercooked ground beef and raw milk being the major vehicles of food borne outbreaks. VTEC has been implicated as an etiological agent of individual cases and outbreaks in developed countries. This study was designed to determine the prevalence of VETEC in diarrheic dairy calves up to 20 days of age in Najaf, Iraq.

**Materials and Methods:**

326 fecal samples from diarrheic calves were collected for isolation of *Escherichia coli* O157:H7 and non-O157 VTEC isolates. Non-sorbitol fermentation, enterohemolysin phenotype, and slide agglutination with antisera were used for screening and detection of these serotypes.

**Results:**

Nineteen (5.8%) non-sorbitol fermenting and 3 (0.9%) enterohemolysin-producing *E. coli* were obtained. Only 9 were agglutinated with available antisera and none of them belonged to the O157:H7 serotype. Three were found to be verotoxin positive on Vero cell monolayers. These included serotype O111 (2 isolates) and serotype O128 (1 isolate). All three VTEC isolates were resistant to ampicillin and streptomycin. Two exhibited adherence phenotype on HEp-2 cells.

**Conclusion:**

*E. coli* O157:H7 serotype is not prevalent in diarrheic dairy calves, and VTEC is not a frequent cause of diarrhea in calves in Najaf/ Iraq.

## INTRODUCTION

Verotoxin-producing *Escherichia coli* (VTEC), including O157:H7, was identified in 1982 as an important human pathogen causing bloody diarrhea or hemorrhagic colitis (HC) which can lead to life-threating sequelae such as hemolytic-uremic syndrome (HUS) and has been reported with increased frequency during the past decade as a cause of human illness (1). VTEC has been implicated as an etiological agent of individual cases and outbreaks in developed countries (2). In developing countries, the situation is different. Although an outbreak of bloody diarrhea due to VTEC has been reported in Cameroon, but it is not recognized as a significant cause of human disease in Bangladesh, India, and Iran (3–6). Although the number of serotypes of VTEC causing human disease is increasing, *E. coli* O157: H7 continues to be the dominant cause of HC and HUS (7).

Dairy cattle have been implicated as principal reservoir of VTEC, with undercooked ground beef and raw milk being the major vehicles of food borne outbreaks (8). Earliest surveys of cattle herds performed in the States of Washington and Wisconsin showed a higher prevalence of this organism in heifers and calves than in adult cattle (9). Other studies in Ontario, Canada found that the prevalence of VTEC in calves (2 weeks to 3 months) was significantly higher than cows (10). Subsequent studies have consistently shown that young animals have the highest prevalence rates, although the youngest animals show relatively low rates. The relatively high prevalence in young animals is consistent with the fact that calves, when infected experimentally with this bacterium, shed the organism for a longer period of time than do older cattle (1).

Extensive efforts have been made to isolate VTEC from cattle in various geographical regions across the world, but there has been no report of VTEC in Iraqi cattle. This study was designed to determine the prevalence of VTEC in diarrheic dairy calves up to 20 days of age in Najaf, Iraq.

## MATERIALS AND METHODS

**Study animals.** Fecal samples, from diarrheic calves, were collected from some private dairy farms in the vicinity of Najaf city, from March to August 2006. Diarrheic calves were divided into three groups according to age. Group 1 comprised calves between 2 weeks to 2 months old. Group 2 comprised calves between 3 to 5 months old. Group 3 comprised calves of more than 5 months of age.

**Sample processing.** Fecal samples were collected by rectal swabs using sterilized cotton-tipped applicators and placed in a tube containing 5 ml of sterile enrichment broth (trypticase soy broth, Oxoid, with 50 µg/L cefixime and 4 mg/L vancomycin) and incubated at 37 °C for 18–24 h (11).

**Phenotypic characterization.** A loopful from the growing culture in enrichment broth was sub-cultured onto sorbitol MacConkey agar for detection of nonsorbitol fermenting *E. coli*, and washing sheep blood agar (tryptose blood agar base, with 0.11% CaCl_2_ and 5% washing defibrinated sheep blood) for detection of enterohemolysin-producing *E. coli* (12). After overnight incubation, non-sorbitol fermenting or enterohemolysin-producing isolates were identified using traditional biochemical tests, including indole, methyl red, voges-proskaur, citrate, urease, and Kligler iron agar. For detection of *E. coli* O157: H7, the bacteria which were identified as *E. coli*, other biochemical tests including cellobiose fermentation, -glucuronidase production, and KCN broth turbidity were employed.

**Serotyping.** *Escherichia coli* isolates that gave the following reactions; cellobiose (-), -glucuronidase (-), and KCN (-), were serotyped by slide agglutination technique using *E. coli* O157, and H7 antisera (Murex Wellcolex, UK). All other *E. coli* isolates were serotyped by slide agglutination technique using monovalent 2, 3, and 4 antisera (Welcome, UK). Positive isolates were then identified by using API 20E (Bio Merieux, France).

**Preparation of bacterial lysate.** Bacterial lysates were prepared as described by O‘Brien and LaVaeck (13). A 100 ml portion of synicase broth medium was distributed in each 250 ml Erlenmeyer flasks, and then inoculated with single *E. coli* colonies grown on trypticase soy agar plates. The flasks were incubated at 37°C for 48 h with shaking (Shaker, Sigma, USA) at 180 rpm. The bacteria was harvested by centrifugation at 10,000 rpm (4°C) for 20 min, washed twice with saline solution, and re-suspended in buffer (3.72 g KCl, 2 g MgCl , 2.42 g tris-hydrochloride, and 1000 ml distilled water, pH 7.4). The cells were disrupted by 3 min. of intermittent sonic oscillation (Sonipred 150, MES, UK). The sonic extracts were clarified by centrifugation at 12,000 rpm at 4°C for 1 h, and sterilized with 0.22 µm Millipore filter (Difco, USA).

**Determination of verotoxin production.** Verotoxin was determined in microtiter plates (Lab-Tek, USA) containing 96 wells with 0.1 ml of cell culture (MEM 199 with Hank‘s salts and glutamine, Flow Lab, UK) supplemented with 10% fetal calf serum (Flow Lab), 100 units/ml penicillin G and 100 µg/ml streptomycin. Vero cells (purchased from Central Health Lab, Baghdad) monolayer were obtained by seeding 1.6 X 10^4^ cells per well, 1 to 2 days at 36°C in 5% CO_2_ incubator (Memmert, Germany) before use. Four-fold dilutions of bacterial lysates in cell culture medium were added in 0.1 ml quantities into wells, and the plate were incubated at 36°C in 5% CO_2_ incubator. Wells containing cells not exposed to bacterial lysates were negative controls. Vero cells were examined daily for 7 days and morphological effects were recorded (14).

**Antibiotic susceptibility testing.** The following antibiotics were studied and were provided by the manufacturer: nalidixic acid, tetracycline, ampicillin, chloramphenicol, carbenicillin, kanamycin, gentamycin, and trimethoprim-sulphamethoxazole. *In viro* susceptibility tests were performed using agar diffusion method on Muller-Hinton agar medium. Results were interpreted according to the recommendations of the National Committee for Clinical Laboratory Standards (15).

**Tissue culture adhesion test**. Adhesion patterns to HEp-2 cells (purchased from Central Health Lab., Baghdad) in culture were assessed as previously described (16). Monolayer of HEp-2 cells grown on cover slips (diameter 13 mm) in 24-well plates (LabTek) were prepared in the absence of antibiotics. Twoday-old monolayers of HEp-2 cells were used for the tests. Bacterial isolates were grown overnight at 37°C in trypticase soy broth. Before the test, monolayers were washed once with Dulbeccos PBS (BDH, UK). One ml of MEM medium without antibiotics or sera was added to each well. The overnight bacterial culture (20 µl) was inoculated into each well and the plates were incubated at 37°C for 30 min. The monolayers were washed 6 times with PBS, and 1ml of the medium was added to each well. After a further 3h incubation period, the monolayers were washed 3 times with PBS, fixed with absolute methanol, stained with 10% (V/V) Giemsa stain (BDH), and examination showed the bacteria adhering to HEp-2 cells.

**Statistical analysis.** The X^2^ test was used for statistical analysis*.* P < 0.05 was considered to be statistically significant.

## RESULTS

Table 1 shows the number of non-sorbitol fermenting and enterohemolysin-producing *E. coli* isolates recovered from the diarrheic calves. A total of 326 diarrheic calves samples were examined for detection of these isolates. Non-sorbitol fermenting *E. coli* isolates were obtained from (5.7%) calves in group 1, (8.7%) in group 2, and (2.7%) in group 3. However, no significant difference was found among the cattle of different age groups (P > 0.05). On the other hand, enterohemolysin positive *E. coli* isolates were detected in age groups 2 and 3 in very low percentages. No enterohemolysin positive *E. coli* isolates were detected in age group 1 of the tested calves. The isolation rate of enterohemolysin-producing *E. coli* showed no significant association among groups of calves tested (P > 0.05).

**Table 1:** Non-sorbitol fermenting and enterohemolysin-producing *E. coli* isolates recovered from fecal samples of diarrheic calves.

Calves age group	No. of calves tested	No. (%) of isolates exhibited	
	
		Non-sorbitol fermentation	Enterohemolysin production
Group 1^a^	87	5 (5.7)	-
Group 2^b^	126	11 (8.7)	2 (1.6)
Group 3^c^	113	3 (2.7)	1 (1.6)
Total	326	19 (5.8)	3 (0.9)

a 2 weeks to 2 months; ^b^ 3 to 5 months; ^c^ more than 5 months.

All case calves excreted watery manure of grayish or yellow color and in 20 samples blood flecks and/or mucous (6.1%) were presented.

***E. coli*** **phenotyping and serotyping.** Biochemical characteristics of the non-sorbitol fermenting (19 isolates) or enterohemolysin-producing *E. coli* (3 isolates) showed that they behave as typical *E. coli* when they grow on the classic screening medium sorbitol MacConkey agar. These isolates were further screened serologically. Only 9 isolates were agglutinated with available antisera (Table 2). Three isolates belonged to serotype O111, 2 isolates to serotype O44, and 1 isolate to each of serotypes O128, O119, O86, and O26. The serotype O157:H7 was not detected in this study.

**Table 2:** Characteristics of 9 *E. coli* serotypes isolated from faecal samples of diarrheic calves.

		No. of isolates exhibited			
		
Serotype	No. of isolates	Non-sorbitol fermentation	Enterohemolysin production	Verotoxin production	calves group
0111:K58 (B4)	2	1	1	2	Group 2
•111:K58 (B4)	1	-	1	-	Group 3
044:K74 (L)	1	1	-	-	Group 1
044:K74 (L)	1	1	-	-	Group 2
026:K60 (B6)	1	1	-	-	Group 1
086:K61 (B7)	1	1	-	-	Group 2
0119:K69 (B14)	1	1	-	-	Group 2
0128:K67 (B12)	1	-	1	1	Group 3
Total	9	6	3	3	

**Determination of verotoxin production.** In this investigation, an attempt was made to evaluate the frequency of verotoxin production in 9 isolates (Table 2). Results demonstrated that the cell lysates of 3 (0.9%) isolates (obtained from 326 diarrheic calves) had the same irreversible cytopathic effect in Vero cell monolayers (Vero cells appeared round, shriveled, and many floated free in the medium). Of the three VTEC; two isolates belonged to the serotype O111 and one isolate belonged to the serotype O128. Two isolates (1.6%) were from calves 3 to 5 months of age (group 2). Results also showed that 2 (66.7%) of the three VTEC isolates were enterohemolysin positive and one isolate was a nonsorbitol fermenter (Table 2).

**Antibiotic susceptibility testing.** The results of the *in vitro* susceptibility to antibiotics of the 3 isolates of VTEC are presented in Table 3. All isolates were resistant to ampicillin and streptomycin. Whereas 2 (66.7%) isolates belonging to serotype O111 were resistant to cephalosporin and tetracycline, one (33.3%) isolate belonging to serotype O128 was resistant to gentamicin. However, nalidixic acid, chloramphenicol, carbenicillin, and kanamycin were the highly effective antibiotics against the VTEC isolates tested.

**Table 3:** In vitro susceptibility of three VTEC isolates obtained from diarrheic calves to antibiotics.

*E. coli* serotype	Susceptibility to antibiotics*								

	AMP	S	CE	TE	G	NA	C	PY	K
0111:K58 (B4)	R^b^	R	R	R	S^c^	S	S	S	S
0111:K58 (B4)	R	R	R	R	S	S	S	S	S
0128:K67 (B12)	R	R	S	S	R	S	S	S	S

* AMP, ampicillin; S, streptomycin; CE, cephalosporin; TE, tetracycline; G, gentamicin; NA, nalidixic acid; C, chloramphenicol; PY, carbenicillin; K, kanamycin.

b R, resistance; ^c^ S, sensitive.

**Fig. 1:**
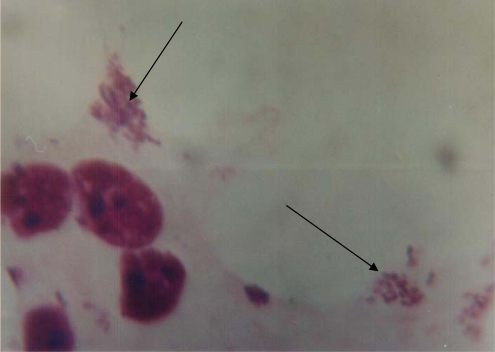
*E. coli* O128 showing localized adherence pattern of attachment to HEp-2 cells (Geimsa stain, X1000).

**Fig. 2:**
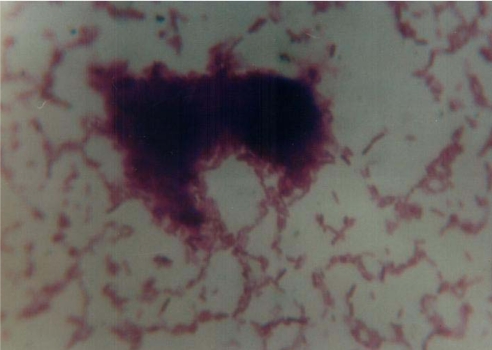
*E. coli* O111 showing aggregative adherence pattern of attachment to HEp-2 cells (Geimsa stain, X1000).

**Adherence of VTEC isolates.** Results of adherence properties of VTEC isolates found that 2 of the 3 VTEC isolates were adherent to HEp-2 cells and that two adherence patterns were detected. In one isolate (VTEC O128), the bacteria were bound to localized areas of HEp-2 cells in which they form very clearcut microcolonies. This pattern is called localized adherence (Fig. 1). In another isolate (VTEC O111), the bacteria were clumped with a characteristic of stacked brick appearance found on the surface of HEp-2 cells and on glass slide free from cells. This pattern is called aggregative adherence (Fig. 2).

## DISCUSSION

The classic screening medium for *E. coli* O157:H7 is sorbitol MacConkey agar. This method exploits the fact that *E. coli* O157:H7, unlike 90% of other *E. coli* isolates does not ferment sorbitol rapidly (17). Other studies reported that sorbitol MacConkey agar medium is a useful, rapid, reliable screening aid for the detection *E. coli* O157:H7 in stool samples, but it is not generally useful for detection of VTEC strains of serotypes other than O157:H7 (18). However, the study of Ojeda *et al.* (19) showed that all 19 VTEC strains isolated from patients with hemolytic-uremic syndrome were sorbitol negative. On the other hand, it has been suggested that the enterohemolytic phenotype, detected on washing sheep blood agar is highly efficient for detection of most of the VTEC strains that are pathogenic to humans and animals (3). Beutin *et al.* (20) found that 89% of 64 VTEC isolates showed a correlation between enterohemolysin and verotoxin production. Results also showed that non-sorbitol fermenting *E. coli* isolates were detected in 5.8% of all diarrheic cattle tested (Table 1). Several investigators declared that more than 5% of *E. coli* isolates were unable to ferment sorbitol rapidly (21).

The inability of this study to detect the serotype O157:H7 among non-sorbitol fermenting and enterohemolysin-producing *E. coli* isolates in cattle confirms the results obtained by other authors, who reported that this serotype is uncommon in cattle and its isolation rates are much lower than those of nonO157:H7 serotypes (8, 22, 23). On the contrary, Wells *et al.* (9) determined the prevalence of *E. coli* O157:H7 among cattle of different age groups and found that this organism was isolated from 5 of 210 calves (2.3%), but only 1 isolate was documented of 662 adult cows (0.15%). Surveys of United States dairy and beef cattle have found *E. coli* O157:H7 in 0 to 2.8% of animals, with the highest isolation rates reported from younger rather than older animals (9, 24). The morphological changes in Vero cell monolayers in this study were the same as that described in other studies (25).

Results of this study revealed that out of three VTEC isolates, two were from calves with 3 to 5 months of age. This result agreed with the finding obtained by Dutta *et al.* who found that 4 (6.5%) of the 61 samples from diarrheic cattle were positive for VTEC and all the positive samples were from calves below 6 months of age (5). Results also revealed that two (66.7%) of the three VTEC isolates recovered from the diarrheic cattle in the present study were enterohemolysinproducers (Table 2). Other studies reported that verotoxin production and enterohemolysin production were closely associated (26, 7). Djordjevic *et al.* (27) showed that 75 (89.3%) of 84 VTEC strains isolated from 1,623 diarrheic sheep in Australia expressed enterohemolysin. On the other hand, Reissbrodt *et al.* (28) found no genetic linkage between VT production and sorbitol fermentation.

The high sensitivity of VTEC isolates in the present study to most of the antibiotics tested may be due to low number of the VTEC isolates obtained. However, all the isolates were resistant to ampicillin and streptomycin. Orden *et al.* reported that *E. coli* strains isolated from diarrheic dairy calves showed low resistance to cephalosporins and quinolones antibiotics (29). It is suggested that resistance to antibiotics has become more prevalent in VTEC isolates. In the United Kingdom, the proportion of 157 VTEC resistant to at least one antibiotic has increased from 10% in 1992 to 20% in 1994 (30).

High adherence capacity has been considered as an important factor for the maintenance of bacteria on the mucosal surface of the host organism. Different studies from various parts of the world (16, 31) incriminated this pattern as virulence factor. Results of this study detected two adherence patterns among VTEC isolates, localized and aggregative adherence patterns. Other studies examined the adherence of VTEC strains. Willshaw *et al.* (32) found that 13 of 48 VTEC human isolates exhibited a localized phenotype on HEp-2 cells. Aslani *et al.* (31) showed that 18 of 70 VTEC isolates manifested difference adherence patterns to HeLa cells. Based on the observations in this study, it can be concluded that the *E. coli* O157:H7 serotype is not prevalent. This study also showed that VTEC is not a frequent cause of diarrhea in calves in Najaf.
